# The influence of cash assistance on the localisation agenda in Kenya’s humanitarian sector

**DOI:** 10.4102/jamba.v15i1.1496

**Published:** 2023-11-21

**Authors:** Pablo V. Holm-Nielsen, Peter Furu, Emmanuel Raju

**Affiliations:** 1 Global Health Section and COPE – Copenhagen Center for Disaster Research, Faculty of Health and Medical Sciences, University of Copenhagen, Copenhagen, Denmark; 2 Unit for Environmental Sciences and Management, African Centre for Disaster Studies, North-West University, Potchefstroom, South Africa

**Keywords:** disaster response, cash and voucher assistance, localisation, Kenya, grand bargain, cash interventions

## Abstract

Cash and voucher assistance (CVA) has gained importance as a modality for humanitarian disaster response during the last decade. Research has documented its benefits and listed challenges for implementation. Simultaneously, humanitarian organisations have committed to the localisation agenda to better serve people affected by disasters through local actors. These two ongoing transformations in the humanitarian sector may support or challenge each other. The authors use Kenya as a case study to analyse how CVA influences the localisation agenda in the humanitarian sector. Semi-structured key informant interviews were conducted with national and international organisations to gain insights on how the international organisations and their local implementing partners view and experience the mutual support or potential tensions between CVA implementations and the localisation agenda. The analysis is based on seven dimensions of localisation applied to CVA in Kenya based on existing frameworks. The findings indicate that CVA can support the localisation agenda if properly managed. It provides smaller organisations an opportunity to get involved without expensive structures. International organisations need to redefine their role and withdraw from direct implementation and be willing to give up power. The local organisations benefit from forming national networks that give them a voice within the humanitarian system. These transformations rely on personal leadership and on capacity development focusing on coordination, collaboration and organisational strengthening beyond the technical skills needed for implementation.

**Contribution:**

This study identifies how CVA and the localisation agenda affect each other in Kenya. This contributes to the understanding of the future development of the humanitarian sector.

## Introduction

Cash and voucher assistance (CVA) is becoming fundamental to disaster and humanitarian response worldwide (Clarke, Stoddard & Tuchel 2018). Cash and voucher assistance is the ‘direct provision of cash transfers and/or vouchers for goods or services’ to the affected population (CaLP Network 2022). Its benefits and challenges have been shown in research and practice (Smith et al. 2018; Spiegel 2015; Tappis & Doocy 2018) as well as in the needed conditions for implementation (Doocy & Tappis 2017; Kreidler & Rieger 2022). It is expected that the modality of aid delivery in disaster response will keep increasing its relevance and significance in the future (Keith et al. 2020) by changing the humanitarian sector and the relationships between humanitarian actors (Harvey & Bailey 2015; Holm-Nielsen et al. 2022). This is crucial in the light of the Grand Bargain (IASC 2019), where humanitarian organisations have committed to the *Localisation Agenda* to serve people affected by disasters in a more context-appropriate manner (Apthorpe & Borton 2019). A definition of localisation has not been agreed upon (ICVA 2018); however, localisation can be described as the respect and strengthening of the leadership of local actors in disaster response (Fabre & Gupta 2017). However, despite some efforts focused on capacity development (Lewis 2021), the relationship between CVA and localisation has not been fully understood or researched. If the commitments of the Grand Bargain are to be achieved in the context of the humanitarian transformation, it is important to understand whether there are contradictions between the design and implementation of CVA programmes and the concept of localisation. This has not been systematically studied leaving a huge knowledge gap. Therefore, the aim of this paper is to analyse the mutual influence of CVA and localisation on each other using Kenya as a case study.

Because CVA implementation and localisation are highly context-specific (Aker 2017), it is relevant to study the interplay between CVA implementation and the localisation agenda in a concrete setting, where the potential of synergies may be present. To study this, the authors used Kenya as a case where many humanitarian organisations (local, regional and international) are present (UNOCHA 2021) and CVA is widely used as a modality for humanitarian response (Haushofer & Shapiro 2013). This provided an ideal setting for studying the relationship between CVA and localisation. The objective of the study was thus to critically examine how CVA interventions and the localisation agenda influence each other in Kenya. In particular, the study determined how the trend to utilise one main international actor or consortium for CVA implementation affects the localisation agenda. To achieve that, the study determined how the international and local organisations (LOs) in Kenya understand and apply the concept of localisation in relation to CVA interventions, analysed the interactions between international and LOs related to CVA implementation in Kenya and examined the extent to which CVA implementation and the localisation agenda influence each other. For the purpose of this study, the analysis was based on localisation frameworks that were adapted to CVA implementation in Kenya. There are endless discussions on the definition of localisation. Here, the authors explore what localisation means to the organisations in Kenya from their perspective. This study focused on the relationship between LOs and international organisations and how CVA influences this relationship. The organisations involved in the study will broadly be classified into International Non-Governmental Organisations (INGOs) with a presence in Kenya or LO. Local organisations refer to Kenyan organisations that work in humanitarian action. It includes national NGOs, civil society organisations, ethnic-based organisations, representatives of pastoralist groups, among others. Further, the term ‘donor’ will refer to the institutional donors from the Global North, even though in the interviews some LOs used the term donor for INGOs or United Nations (UN) agencies.

Kenya provided ample opportunities for learning as both CVA and localisation efforts are well established in the context of humanitarian response (Atputharajah 2020; Odera 2017; Shapiro & Haushofer 2016; Tiwari et al. 2016). Hence, the relationship of CVA and localisation could be studied minimising the disturbance caused by either area not being effectively implemented, as may have been the case in other contexts. Hence, the study did not have to address potential improvements in either CVA or localisation implementation and could concentrate on their mutual influence. Further, Kenya is routinely affected by a variety of slow-onset and rapid-onset disasters such as droughts, heatwaves, floods and refugee crises of the Horn of Africa (Rudari, Conijn & De Angeli 2018). In recent years, the country has also been affected by locusts (Kimathi et al. 2020) and the coronavirus disease 2019 (COVID-19) pandemic (Karijo, Wamigu & Boit 2021). To these crises, the response focuses on programmes for nutrition, livelihoods or basic needs, among others (Rudari et al. 2018). Cash and voucher assistance is commonly used as a response modality and mobile money is often used as a mechanism through a national network operator (Harvey, Sossouvi & Hurlstone 2018). Kenya is also a country where innovative CVA implementation has been piloted (Oliveros 2018). In Kenya, like in other contexts, there is a trend by donors to concentrate CVA programmes in a consortium of INGOs, arguing increased cost efficiency (Holm-Nielsen et al. 2022). It has been questioned whether this trend works against the localisation agenda (Lewis 2021). Specific programmes for localisation efforts have also been active for some years (Start Network 2015) focusing on the self-assessment of LOs and contextualising indicators for localisation (Baguios et al. 2021). Compared to neighbouring countries, Kenya is characterised by having experienced LOs with a high capacity for implementation (Kabetu & Iravo 2018). Analysing the relationship between CVA and localisation in the context of Kenya can provide valuable insights for both researchers and practitioners in the humanitarian sector, and potentially these may be applied to other countries or regions (Ruddin 2016).

## Cash assistance in a localisation context

The aim of this section is to provide a brief overview of the overlaps between the two approaches namely, CVA and localisation and the efforts that have taken place in making linkages between them. Numerous studies have revealed the benefits, challenges and opportunities of CVA in humanitarian action (Bailey & Harvey 2015; Peppiatt, Mitchell & Holzmann 2001), showing that the dignity of the affected population is enhanced (Venton, Bailey & Pongracz 2015) and that it is a cost-effective way to make the aid arrive to the people in need (Tappis & Doocy 2018). This was accompanied by discussions on appropriateness (Farrington, Harvey & Slater 2005), the correct way of implementing CVA (Creti & Jaspars 2006; Farrington & Slater 2006) and the conditions that need to be in place, such as the functioning markets (Kopczak & Matthews 2016). The Background Note for the High-Level Panel on Humanitarian Cash Transfers asked the specific question ‘what is the role of cash in localisation?’ (Harvey & Bailey 2015) which has not yet been fully answered making a compelling case for this study.

Studies have expressed concern that the current and future models of CVA implementation may work against the localisation agenda (Lewis 2021) and that innovation in CVA approaches may be limited by the conformity of humanitarian organisations (Monich, Holm-Nielsen & Raju 2023). Reasons include the unification of CVA implementation into one single agency or consortia (Holm-Nielsen et al. 2022), the technological divide that the development of fintech may introduce in the future (Doocy & Tappis 2017), the unbalanced level of capacities between international and LOs when it comes to CVA programmatic design (Lewis 2021) and the overwhelming power of international organisations and companies compared to its national counterparts (Bennett, Foley & Pantuliano 2016; Haakenstad et al. 2018). For this reason, the study of the influence of CVA on localisation has to rely on the existing analysis of the operationalisation of localisation.

Many methodologies for analysis of localisation have been developed (Konyndyk & Worden 2019). The exact meaning of localisation has not yet been defined (ICVA 2018). There have also been discussions on who is the ‘local’ (Mac Ginty 2015). For this study, localisation can be described as:

[*A*] process of recognising, respecting and strengthening the leadership of local authorities and the capacity of local civil society in humanitarian action, to better address the needs of affected populations and to prepare national actors for future humanitarian responses. (Fabre & Gupta 2017:1)

The main commitments from the localisation agenda address issues such as financing, partnership, capacity strengthening, coordination, recruitment and communication (Charter4Change 2019). At the Grand Bargain, many donors and humanitarian organisations committed themselves to making humanitarian response as local as possible, for example by channelling up to 25% of the funds directly to local and national actors by 2020 (IASC 2019). This goal has by no means been achieved (ANALP 2021). It has further been argued that the indicators of the Grain Bargain workstream on localisation are not optimal for the desired purpose (ICVA 2018). Instead of measuring capacity development or direct funding to LOs, what is important is how relationships between organisations are created or developed (ICVA 2018).

There have been attempts to regulate relationships between the people affected by disasters and the humanitarian agencies by making the affected population part of decision-making and held the agencies accountable for the decisions they make on behalf of the affected population (Barbelet 2018). However, research has shown that the people most affected by crises have the least influence in the humanitarian decisions (Collison 2016).

The mutual influence of CVA and localisation is hence relevant for the future of humanitarian aid delivery. The study of this relationship was based on the different components of localisation operationalised in a framework highlighted in the next section.

## Conceptual framework for localisation and cash and voucher assistance

Research criticises that power is held by the international humanitarian system centred around the UN (Konyndyk & Worden 2019) and that international organisations keep the primary relationships with the donors (Barnett & Walker 2015). Even though the importance of local actors has been repeatedly acknowledged (Apthorpe & Borton 2019), evaluations have revealed a lack of implementation in practice (Collison 2016). Further, the needs of the affected population emphasised in disaster planning are primarily related to the sectors of the major agencies (Konyndyk 2018), showing that the strategies of international humanitarian actors have more prominence than the local actors (Barnett & Walker 2015). Some of the reasons are that the competition for funds promotes self-interested growth and disincentivises transfer of power to potential competitors for those funds (Collison 2016). Even in situations where collaboration is preferred, the organisational motivation is towards larger funds from donors (Ramalingam 2014).

Donors have indicated difficulties in channelling funds directly to LOs due to a lack of absorption and monitoring capabilities. Therefore, their preference is to work with a large partner on a localisation approach (Holm-Nielsen et al. 2022). Further, an important aspect of localisation is capacity development (Lewis 2021). It has been argued that capacity development should be based also on what the LO believes they need and want. In many cases, the INGO decides what LO needs, or worse, they base the trainings only (or mainly) on the training experience that the INGOs already have (Hagelsteen, Becker & Abrahamsson 2021).

Several frameworks for localisation have been developed for analysis and implementation of localisation compared in Table 1. The Global Mentoring Initiative (GMI) defines seven dimensions of localisation (Van Brabant & Patel 2018), the Overseas Development Institute (ODI) defines three dimensions and five levers for localisation implementation (Baguios et al. 2021), a consortium of INGOs defines four pathways to localisation (Accelerating Localisation through Partnerships 2019) and the Network for Empowered Aid Response (NEAR) developed a localisation performance measurement framework based on six components (Featherstone 2019). The authors have combined the different frameworks into eight areas that specifically focus on the interaction between CVA and localisation. These areas served as basis for the data gathering presented in the results section.

**TABLE 1:** Comparison of frameworks for localisation with the eight areas for localisation developed for studying the interaction between cash and voucher assistance and localisation.

The GMI	ODI	INGOs (Christian Aid, CARE, Tearfund, ActionAid, CAFOD, Oxfam)	NEAR	Framework for localisation applied to CVA in this study
Capacity	Knowledge	Capacity	Capacity	Capacity development
Coordination mechanisms	Relationships	Coordination	Coordination and complementarity	Coordination mechanisms
Partnerships	-	Partnerships	Partnerships	External relations
Funding	Resources	Financial resources	Funding	Funding
Participation revolution	Agency	-	-	Trust
	ways of being	-	Participation	Power
Policy	Priorities	-	Policy, influence and visibility	Programme design
	Decision-making	-	-	-
Visibility	Delivery	-	-	Visibility

GMI, Global Mentoring Initiative; ODI, Overseas Development Institute; INGO, International Non-Governmental Organisations; NEAR, Network for Empowered Aid Response; CVA, cash and voucher assistance.

Each of the four existing frameworks was reviewed comparing the concepts that were included in the particular framework. The underlying concepts use slightly different nomenclature to point at similar areas related to localisation. For example, the term ‘capacity’ in three of the frameworks was labelled ‘knowledge’ in the ODI framework but covered similar concepts related to capacity development of the LO. Similarly, ‘relationships’ covered similar concepts as ‘coordination’ and ‘partnerships’. The comparison of the different dimensions of localisation or pathways to localisation allowed us to extract eight relevant areas of localisation related to CVA. Table 1 presents the comparison of the four frameworks for localisation available in literature along with the conceptual framework developed for this study based on previous studies.

Table 2 presents an overview of the eight areas with an indication of the aspects of CVA implementation that are included in the area. Capacity development of CVA related to localisation describes the process of decision-making on what trainings are relevant for the LO and whether organisational development is part of this decision process. Coordination mechanisms describe the activities related to coordination of CVA programmes among LOs and between LOs and INGOs and various levels in the Kenyan humanitarian sector. External relations refer to the access to donors by LOs and the relationship to financial service providers (FSP) supporting the programmes. Funding covers the access and bureaucratic barriers to funding and the sharing of the indirect costs that the programmes include. Trust includes the relationship between LOs and INGOs with regard to compliance and the perception of risk of corruption. Power describes the imbalance of the relationship between LOs and INGOs as well as the space given to LOs in the Kenyan humanitarian sector. Programme design addresses the initial stages of the programme related to the decision-making about the programme, the character of the partnerships that are established and the respect for the strategies of LOs, INGOs and donors. Finally, visibility describes the extent to which LOs are given credit for their activities in reports and in the international fora. These eight areas of localisation applied to CVA next to the understanding and application of localisation for each organisation are used as the basis for interviews and guide the analysis of the data.

**TABLE 2:** Areas of application of localisation with an explanation of what is covered by the area with regard to cash and voucher assistance implementation.

Area	Aspects of CVA included
Capacity development	Decision on trainings
	Organisational development
Coordination mechanisms	Coordination of national actors
	Coordination with international actors
External relations	Relations to donors
	Relations to FSP
Funding	Access and bureaucracy barriers
	Indirect costs
Trust	Compliance and corruption
Power	Power imbalance
	Relationships to sector
Programme design	Decision-making
	Partnerships
	Strategies
Visibility	Giving credit to field implementation

CVA, cash and voucher assistance; FSP, financial service providers.

## Research methods and design

This study is based on qualitative research methods. Data were collected in 2022 using semi‑structured key informant interviews (DeJonckheere & Vaughn 2019). In total, 15 respondents were interviewed. The respondents were selected purposely for having experience in CVA implementation during disasters in Kenya, either from INGOs or LOs in order to be able to represent several types of organisations and points of view. Thus, insights were gathered from larger international organisations and agencies (some of which were part of a CVA consortium) and respondents representing LOs (some of which were part of a national network of LOs). In one case, the LO was directly funded by a back donor from the Global North. The respondents are shown in Table 3 along with the codes used to present the findings.

**TABLE 3:** Background of respondents in the study and corresponding codes used as a reference to statements in the findings section.

Respondents	Member of consortia or network	Not member of consortia or network
INGO	3	3
	(F, G, H)	(A, I, J)
LO	6	3
	(B, C, D, K, L, N)	(E, M, O)

INGO, International Non-Governmental Organisations; LO, Local Organisations.

Initially, the respondents were identified as members of the national cash working group (NCWG) co-chaired by the Kenyan Red Cross. Each of those respondents was asked to identify who their collaborating partners were for CVA programmes. Purposive snowball sampling (Naderifar, Goli & Ghaljaie 2017) was used to select further respondents who could provide relevant experience, insights and knowledge. All the respondents were identified based on their experience with the phenomenon under study (Holzhauser & Cresswell 2008), namely, CVA implementation in disasters in Kenya and could therefore answer questions about CVA and localisation in Kenya.

The interview data were recorded, transcribed and analysed by coding and highlighting important statements, emerging issues and concepts (Kvale 2011). The codes were based on the dimensions of localisation and the sub-codes developed during the analysis process identifying themes that respondents referred to. The themes that emerged from the analysis revealed thematic areas that are essential to the interaction between CVA and localisation in Kenya’s humanitarian sector, which will serve as basis for the discussion.

Anonymity was ensured for individuals and organisations allowing an open discussion (Dearnley 2005). The semi‑structured interview guide included questions on the respondents’ experience in CVA in Kenya, their understanding of what localisation is and how they think CVA influences localisation. Then, each of the seven dimensions of localisation presented in the framework for localisation (Table 2) was discussed in the light of CVA implementation in Kenya. The semi-structured nature of the interview meant that the actual questions were adapted to background and the level of expertise to which the respondent pertained. The respondents all had a vast experience in the humanitarian system and in CVA implementation in Kenya, giving them the possibility of a more generalised view on the topic; thus, helping us indicate analytical generalisations (Flyvbjerg 1997, 2011).

### Ethical considerations

Ethical clearance to conduct this study was obtained from the Masinde Mulior University of Science and Technology Institutional Ethics and Review Committee (IERC). (No. MMUST/IERC/041/2022).

## Findings

This section firstly focuses on the understanding on localisation and CVA in Kenya by the respondents and the effect of CVA on the creation of networks of organisations and its importance in the localisation in Kenya. Secondly, the presentation of the findings explores each of the areas of localisation presented in the framework and its relationship to CVA.

### The Kenya story on localisation and cash and voucher assistance

All respondents agreed that the definition of local actors is dependent on the point of view of the national organisation in the capital, who may consider an organisation in a province as local. A basic characteristic of an LO was identified as their knowledge of the affected communities and the fact that they have a permanent presence in the same context.

All respondents from LOs emphasised that localisation is based on their greater knowledge of the context and understanding of local processes. In their view, localisation means to respect decisions made at county-level. Although respondents from LOs reported to have experienced a focus on localisation in the past years, they felt that there is still room for improvements. International non-governmental organisations, on the other hand, indicated that localisation is more than capacity development and responding through an LO. ‘It is about integrating that LO in the design and decision-making’ (G). To the INGOs, localisation is a shift of mentality which re-defines their role. ‘It is about reorientating your systems and withdrawing’ (H). It was suggested that there is a need for a radical change instead of incremental changes as have taken place until now. Communities and the local private sector were pointed out as missing from the general localisation discussion. However, the concepts of CVA and localisation are shown to be well established in Kenya. There was a general consensus among respondents that CVA implementation has helped localisation in Kenya. ‘CVA relies on the skills that the LOs already have’ (H). Local organisations further indicated that CVA helps localisation because it strengthens their relationship to the recipients of aid and it has simplified the delivery process. Several respondents mentioned that during the COVID-19 pandemic, there was an activation of CVA and localisation, due to LOs having a larger access to the communities and CVA being preferred due to lower risks of infection. ‘It was a hopeful moment for LOs, which unfortunately, disappeared after the lock-down’ (G).

#### International consortia and local networks for cash and voucher assistance implementation in Kenya

In Kenya, a consortium of INGOs for CVA was created 4 years ago, called the Kenyan Cash Consortium (KCC), which works closely with a network of LOs called the Arid and Semi-Arid Land Humanitarian Network (AHN). Arid and Semi-Arid Land Humanitarian Network was formed, pushed by the initiative of individuals who had international experience but chose to work locally in Kenya. The aim of AHN is to ‘influence the localisation agenda by finding likeminded people who shared a long term vision’ (K). Some INGOs took strategic and financial risks to strengthen the emergence of the network of local partners in the Arid and Semi-Arid Land (ASAL) counties, which was crucial for the development of AHN.

All respondents from AHN indicated that the creation of the network was greatly influenced by the implementation of CVA programmes that did not require a logistics set-up. Respondents indicated that AHN lets LOs have more strength in the humanitarian system. ‘We have bargaining-power and a strong platform for fighting for our space’ [L]. Other benefits of AHN include having common tools and approaches, having a platform to raise issues and fight for the communities they represent. The creation of the AHN was reported to have a secondary effect of inspiring other networks to be created. Two LO respondents [K, N] explained that county-level networks of LOs are starting to appear.

A few respondents also indicated that INGOs benefit from the existence of AHN, given that they have a larger geographical presence in the country and have introduced a harmonised way of assessment and reporting. One critical LO was of the opinion that AHN benefits the INGOs more than the LOs, giving KCC the opportunity to show localisation to donors.

#### Framework for localisation

The seven dimensions of localisation presented in Table 2 are analysed in the context of CVA implementation in Kenya. Some of the dimensions are combined when the respondents expressed a strong relationship between them.

#### Capacity development

Capacity development is defined as ‘the process of developing and strengthening the skills, instincts, abilities, processes and resources that organizations need’ to engage in disaster response (UNAI 2023). There are different approaches to capacity development in Kenya based on the level of applied localisation. A few respondents indicated that often costs for capacity development are given to INGOs that prepare a training, instead of going to the LO for organisational development. ‘Therefore, capacity development is perpetual’ [E]. Capacity development is generally not handed over to LOs, like fieldwork activities are.

In the relationship between KCC and AHN, a renewed capacity development approach focuses on LOs becoming independent of INGOs. It relies on strengthening capacities in the management level on strategy and organisational development. ‘The aim is understanding how the international humanitarian system works, how to navigate the politics and how to access donors’ (B). Although there have been trainings in programme and consortium management in Kenya, a significant change of the training plans has not yet become a reality. A respondent from an INGO (D) pointed out that the shift in the aim of capacity development demands a shift in the profiles that deliver the trainings.

It was also pointed out that good capacity development often leads to staff leaving the LO for higher international salaries. The local humanitarian system would need a long-term mentality from experienced and motivated national staff. ‘As you decide to take the local role, it is more about passion and motivation than a salary’ (K). This also reflects a disparity in pay structures between different levels of organisations.

#### Programme design

Programme design in the context of this study refers to the definition of the programmes that will address the needs of the affected population. Opinions and reflections were most diverse when discussing dimensions of programme design in localisation. Some LOs (M, E) indicated that they should be more involved in programme design, explaining that INGOs and donors decide what they expect the programme to include. These LOs complained that the selected locations, modalities and sectors are the ones that fit the strategy of INGOs and donors, more than the needs that are identified by the assessments. A different point of view was that the INGOs do engage the LOs at the initiation of the project and in the proposal writing. They stated that LOs have an influence in the identification of the locations and in the design of the response. A member of AHN (L) reported that project proposals and definitions can even be created with a bottom-up approach, where the LO submits proposals to the INGO that further it to the donor.

A disagreement in CVA programme design between INGOs and LOs concerns the balance between coverage and impact. The LOs wish to spread the CVA help more widely to help more people even though it may reduce the transfer value to individual families. The INGOs, on the other hand, believe that this strategy will not have a significant impact and would therefore prefer to reduce the number of targeted villages. It was suggested that one way to alleviate this issue would be to have more predictable funding that would allow for long-term planning. However, the biggest criticism to the CVA programmes in Kenya was the lack of long-term strategies, where the aid is delivered intermittently for a couple of months, depending on the available funding. The LOs questioned the real impact of this approach and argued that it undermines their relationship with the population.

#### Power and trust

All respondents highlighted that trust and power relations greatly influence each other. Cash and voucher assistance programmes in Kenya were described as ‘contributing to maintaining the existing division of power’ (C) in the cases where the INGO and the donor negotiate the conditions of the programme before engaging the LO. Respondents were unanimous in saying that INGOs need to give up power for localisation to be successful. However, the general view suggested that for the transfer of power to happen, it depends on the headquarters in the Global North and the individual managers of the INGOs in Kenya.

All the respondents indicated that the creation of AHN has increased the power of the members of the network, giving them a more prominent role. One risk raised for AHN, was that as the network grows larger, internal agreement becomes more difficult, which weakens the negotiating capacity as a whole network.

All respondents agreed that trust is a big barrier for localisation and for accessing power. However, ‘trust and compliance really mean corruption and fraud’ (C). Respondents indicated that some donors and INGOs view fraud as a real risk with LOs. Further, LOs explained how reported cases of fraud affected the reputation of all of the organisations of the country and strengthens the reasoning for the INGOs to be grant managers. Two respondents (C, F) expressed frustration of the fact that INGOs are more trusted even though cases of fraud have also been reported at that level. It appears that the staff implementing CVA programmes have a similar background in INGOs and LOs. Conversely, several respondents indicated that fraud mostly depends on the top management of the organisation, which enforces or ignores different policies and guidelines. Cash and voucher assistance implementation, however, was reported to lead to enhanced financial controls because it is easier to monitor than in-kind aid.

#### Funding

Respondents indicated that in more traditional CVA programmes in Kenya, funds are given to INGOs that subcontract LOs for implementation. However, in one case (M), the LO received funds directly from donors, indicating that it happened because of proven organisational strength and effective financial control measures.

Funding and the sharing of overhead were identified as barriers for LO organisational development. Local organisations indicated that there has been an improvement in later years, and INGOs explained that further improvement is expected in the future. However, even in the cases where respondents of INGOs were committed to sharing the overhead and indirect costs, they indicated that it depends on the attitude of the donor and on the headquarters of the INGO in the Global North. One INGO [F] indicated that they use the overhead as an incentive for the LOs to deliver financial reporting on time.

Members of AHN explained that they negotiated the overhead as a group. It was an important step for localisation that was possible because of the strength that AHN offers. However, in the AHN consortium, there was reportedly some competition for programmes and funding. The funds that are transferred to the affected population could be transferred directly by the INGO to targeted people through the national FSP. However, in the KCC and AHN relationship, the LOs perform the transfers because showing that larger funds have been managed successfully by the LOs may be important for future programme applications.

#### Coordination mechanisms

All respondents identified that localisation of CVA coordination in Kenya is challenging. Major issues mentioned included competition among INGOs and poor follow-up on processes handed over to the government. Two levels of coordination of CVA programmes were identified namely at national level with the NCWG chaired by the National Drought Management Authority and co-chaired by the Kenyan Red Cross and at county level, with Steering Groups (CSG) and the County Technical Working Groups (CTWG) that refer to them.

Respondents were unanimous in noting that the LOs are notoriously absent from the NCWG. The reasons included that the LOs focus on their own county, the difficulty of travelling to the capital for meetings, that the LOs may not see the value of NCWG for them or that they have not been particularly targeted for participation. During the COVID-19 lockdown, meetings were held online, meaning that participation was no longer restricted to organisations with a physical presence in Nairobi. There were a variety of opinions on whether this under-representation of LOs at the NCWG was a cause of concern. One set of respondents indicated that INGOs represent their LO partners and at a later stage inform them about the outcomes. It was argued that the LOs could give feedback on certain issues to the NCWG through their partner INGOs. Another set of respondents said that LOs should be more present at the NCWG meetings because they could give a better picture of the local context. All of these indicate a weak localisation process in including different partners at the county level in coordination mechanisms.

At county level, LOs are usually members of CTWG and CSG. Some LOs are chairing the CTWG, although several LOs indicated that they required enhanced capacities for that role. At CSGs there are representatives of LOs, INGO, UN agencies and governmental representatives. ‘It is the only platform where all actors are around the same table’ (M).Respondents indicate the need for strengthening the link between the NCWG and the CSGs. One challenge is that the INGOs coordinate among themselves and only with the LOs that are funded through their means, and not with a broader set of national or county-level LOs. A second challenge relates to disagreements on the transfer value that has previously been presented in the programme design sub-section.

#### External relations and visibility

Most respondents linked external relations to the visibility of the organisation’s activities. The general consensus among respondents was that in Kenya usually LOs get the credit for their work in reports and assessments alone. There were exceptions where INGOs had taken credit for data produced by the LOs. Kenyan Cash Consortium members indicated that they are pushing the LOs to be present at international meetings. It was indicated that both KCC and AHN could use each other for advocacy and visibility. Kenyan Cash Consortium represents AHN raising issues at national or international level, while AHN is able to criticise the national institutions more freely if needed.

Respondents critically highlighted that visibility is only promoted when it is useful for the INGO. Respondents from INGOs saw the benefit of giving credit to the LOs, which strengthens funding applications. However, even when INGOs are willing to give the LOs a prominent position towards the donors, it is still the INGO doing most of the work in the applications. The reasons included quality of the application and the capacity for strategic communication to donors. The LOs unanimously felt that they should have direct contact with the donor to discuss the strategy of the CVA programme directly. However, most respondents from the LOs indicated that in the past years there has been a positive strengthening in the relationship between LOs and donors. Respondents from LOs also pointed out that localisation issues are furthermore relevant to the relationship within and between Kenyan organisations. Some of the tensions related to visibility that are relevant in the relationship between INGOs and LOs were replicated among Kenyan organisations. Finally, some of the organisations that have a presence at county and sub-county level felt that they are competing with the larger national Kenyan organisations.

All the dimensions of localisation presented here have an influence on each other and represent a particular contribution to the localisation agenda for the implementation of CVA programmes in Kenya. These aspects will be discussed in the next section.

## Discussion

The findings highlight significant relationships between CVA implementation and the localisation agenda in the Kenyan humanitarian context. The discussion will address this interaction as well as the significance of the creation of networks of organisations and the role, identity and leadership in humanitarian organisations in their influence on CVA and localisation. These topics were identified by the respondents and the subsequent analysis of the data as relevant to the interaction between CVA and localisation in Kenya’s humanitarian sector.

### Localisation and cash and voucher assistance in Kenya

The authors’ research shows that localisation is a relative term, which should not have an absolute definition (ICVA 2018; Mac Ginty 2015). Each organisation defines ‘local’ as whoever is closer to the people receiving the aid – from the Global North to the affected nation, from the capital to the counties, and further to the villages. Therefore, localisation will be any action empowering the next step closer to aid delivery. This is reflected in Kenyan LOs perceiving similar localisation issues with the INGOs as country-based LOs perceive them with regard to national Kenyan organisations.

The findings in this study indicate that localisation in CVA in Kenya is in a continuous process of development and that the humanitarian system needs for a radical change instead of incremental changes as have taken place so far (Holm-Nielsen et al. 2022). While there are changes, there is significant scope for improvements (Bennett et al. 2016) and power relations are still largely unequal (ICVA 2018; Konyndyk & Worden 2019). Several of the areas of localisation from the framework show positive outcomes, especially visibility, sharing of indirect costs and fund management. From a situation where INGOs did not trust LOs, the relationship is slowly moving towards LOs being able to question and challenge INGOs and have a real influence in CVA implementation. However, relations to donors and influence in programme design are still areas that need improvement (Collison 2016). Some LOs also question whether particular INGOs use localisation for their own promotion towards donors, more than giving a more prominent role to their LO partners.

The findings show a variety of opinions from the Kenyan LOs on the relationship with INGOs. Some LOs appreciate the cooperation with INGO because of support for proposal development and in fund raising and see an opportunity to develop and expand (Lewis 2021) especially considering the expected future transformation of the financial sector and its influence on CVA (Monich et al. 2023). Conversely, other LOs are of the opinion that if they had access directly to donors, they would be able to implement the CVA programmes without the INGO, thus lowering the administrative costs (Barnett & Walker 2015). Among the INGOs, there is a variety in opinions on whether this would be the case. Ultimately, the strategies of donors and INGOs for CVA tend to overshadow the strategies that the LOs may have, confirming the criticism found in literature (Collison 2016).

The authors’ research confirms that trust is an important issue for localisation and that cases of corruption and fraud greatly influence that trust (Ramalingam 2014). The implementing staff has the same profiles in INGOs and LOs, so the greatest influence on fraud was shown to be the attitude of top management in the organisations. Capacity development on organisational management would therefore be needed in particular cases.

An important factor for CVA coordination is that LOs are more active at county level than at national level. Local organisations are not active in the NCWG for various reasons, but they participate and even chair the county-level CSG at CTWG. The counties were shown to be the only forum where all actors are present simultaneously.

During COVID-19, there was an activation of localisation and CVA given that LOs had more access to communities because of movement restrictions. Meetings were also held online, giving easier participation to organisations without presence in the capital. However, the momentum was lost after the pandemic, showing that localisation is more dependent on willingness than on opportunity.

### Cash and voucher assistance localisation and networks

The authors’ research shows that CVA helps localisation in various ways. A small organisation can contribute to the programmes without the need of an expensive setup. Cash and voucher assistance relies on the capacities that the LOs already have in community engagement and understanding of the context (Charter4Change 2019). Also, transfer funds can be given to LOs to distribute to the communities in an easier way than it would be with in-kind aid. Hence, showing the capacity of the LO to manage funds (Haakenstad et al. 2018), which is important for future applications and donor relations.

In Kenya, there is an ongoing discussion between LOs favouring the increase of coverage with less funds per family and INGOs preferring to increase impact with larger funds to less families. The discussion is positive for localisation showing the more equal nature of all organisations (Konyndyk & Worden 2019). The discussion is also possible because CVA allows for the grant to be reduced in arbitrary smaller sizes. However, the flexibility of LOs and CVA also allows for short-term implementation without a long-term strategy. In some cases, LOs complained that the aid was distributed over time in an unpredictable way that is dependent on funds more than on strategy.

The authors’ research shows that INGO consortia for CVA are not an impediment for localisation if the rest of the conditions for localisation are correctly managed. A unified CVA entity makes sense for CVA to scale. However, it is important to pay attention to the relationship between the lead actor and the implementing partners. For localisation, it is not so important how many LOs are engaged as how those relationships are created and developed. Hence, some of the indicators for localisation that are used in the Grand Bargain workstream (IASC 2023) were shown not to be optimal.

The authors’ research shows that the local networks of LOs are the most important reason for localisation in Kenya. It gives LOs bargaining power, influence and a voice in the humanitarian system. For localisation to develop, it is important to create the spirit of a national movement among LOs, fostering internal cooperation and trust in their common capacity. However, the cooperation mechanisms in the local networks are not always smooth and are still being defined and their internal processes being consolidated. Therefore, capacity development needs to focus on the creation and coordination of these local networks (Hagelsteen et al. 2021), raising the question of whether the INGOs are well equipped for supporting the development of such capacities.

The creation of AHN was due to the capacity and willingness of individuals with international experience that chose to work locally in Kenya. The creation of the local network was further reported to be possible due to CVA implementation. The vision was strengthened by the support of INGOs that were willing to hand over responsibilities to capable LOs. Secondment of staff by the INGOs was shown to increase the understanding of the challenges and contexts LOs are facing.

### Role, identity and capacity

The findings in this study indicate that localisation in CVA programmes requires that the engagement between INGOs and the LO should be long term and strategic, and not based on single projects or single responses. Protracted crises, like the recurrent droughts witnessed in Kenya (Houldey 2019) give the opportunity for such relationships to be established. Furthermore, localisation in CVA implementation relies on a re-definition of the roles and identities of the organisations involved. This in turn affects the capacity development efforts that are taking place. Capacity development should focus on organisational strength, on cooperation among LOs as well as on technical matters like CVA implementation. However, this type of capacity strengthening requires new profiles giving the trainings beyond technical knowledge on CVA implementation. Also, the humanitarian sector has been shown to be based on poor coordination (Spiegel 2017), which raises the question on how INGOs can contribute to increased capacity in something they themselves are not good at.

The authors’ research shows that in Kenya, localisation in CVA programmes is successful because several INGOs have re-defined their role and with that, their identity. These INGOs are no longer direct implementers but have defined themselves as grant managers. Local organisations perceive that there will be a role for INGOs in CVA implementation in Kenya channelling funds from donors, monitoring that systems and SOPs are functional and that accountability is ensured. Thus, ultimately helping the LOs to become self-sufficient. Ideally, the LOs should help define what the role of the INGOs should be.

The authors’ research further shows that several of the important factors for localisation in CVA programmes rely on individual leadership. Managers in INGOs should be willing to give up power for themselves and their organisations, which may go against their personal interest or the willingness of the HQ of their organisation in the Global North. Kenyan professionals also have the opportunity to choose to work locally. Thus, strengthen LOs and influence the localisation agenda and CVA implementation from the local humanitarian system. They should be encouraged to find other local professionals who are willing to influence the process with a long-term goal in mind. However, this vision requires funding. Creation of networks and national coordination demands resources, and furthermore, for national professionals with experience to have an interest in the creation of a local networks of LOs, the salaries in those positions should be comparable to the ones in INGOs.

## Conclusion

In studying the interplay between CVA implementation and localisation in Kenya, CVA was identified as a key aspect supporting localisation, given that LOs can engage in the response without expensive setups and can rely on their existing capacities and relationships to the communities. The flexibility of CVA enables a discussion on the balance between coverage versus impact. However, the same flexibility may also allow for short-term implementation of aid without proper strategy. The study highlights the existing challenges of defining the concept of localisation, showing that the concept of the local is a relative defined from the position of each actor.

The analysis shows that localisation in CVA implementation relies on a re-definition of the identity and role of INGOs and LOs. The study highlights an increased effort for localisation in great part due to the creation of a national network of LOs that has given a voice to the LOs. Further, there are good examples of willingness of certain INGOs to give up power and withdraw from an implementation role. It is clear that these initiatives require decisions that may collide with the personal interests of humanitarian professionals. In Kenya, a few LOs seem to be finding their space in the county response context. They participate and even lead the county steering committees and technical working groups on cash interventions. They influence the targeting and the response design (in some cases challenging the NCWG and INGOs). Further, they have a direct contact with the affected population they represent.

The authors’ research shows that CVA programmes have facilitated the creation of networks of national organisations. However, it requires capacity enhancement that goes beyond technical skills and needs further organisational development. Funding is needed for capacity development and for the creation, management and maintenance of the LO network. Donors should therefore consider funding the creation of national consortia or networks of LOs. This would foster a long-term relationship between INGOs and LOs, thus strengthening localisation based on CVA implementation. Finally, the research shows that based on a re-definition of roles and an appropriately managed relationship between INGOs and LOs, CVA may be a solid base to build a practical implementation framework for localisation.
